# Social Media for Nutrition Education—A Randomized Controlled Trial to Promote Fruit and Vegetable Intake in a University Setting: “The University of Valladolid Community Eats Healthy” Study

**DOI:** 10.3390/nu16091308

**Published:** 2024-04-26

**Authors:** Laura Carreño Enciso, Beatriz de Mateo Silleras, Sandra de la Cruz Marcos, Paz Redondo del Río

**Affiliations:** 1 Department of Nutrition and Food Science, Faculty of Medicine, University of Valladolid, 47005 Valladolid, Spain; bmateo@uva.es (B.d.M.S.); sandra.cruz@uva.es (S.d.l.C.M.); paz.redondo@uva.es (P.R.d.R.); 2 Spanish Society of Community Nutrition (SENC), 08029 Barcelona, Spain

**Keywords:** intervention, fruit, vegetable, behavioral theory, university, social media, nutrition education, virtual campus, Facebook, Instagram

## Abstract

Social media- and internet-based interventions are nowadays widely used tools in health interventions. Although evidence of their effectiveness is still low, their applications could be very promising due to their affordability and wide reach. The current paper aims to evaluate the effectiveness of an intervention program, “The University of Valladolid Community Eats Healthy” (UVEH), to increase fruit and vegetable (FV) intake in adults from the University of Valladolid (U. Valladolid) employing three online methodologies. A sample of 211 volunteers was randomly assigned into four groups: virtual campus (VC), Facebook (FB), Instagram (IG), and control. An intervention based on the Theory of Planned Behavior was implemented for seven weeks. Data were collected at the beginning (T0) and the end of the program (T1). The Predimed questionnaire was employed to assess FV intake. Vegetable intake was statistically significantly higher in the VC group (17.4% pre vs. 72.7% post). In the rest of the groups, there was also an increase in intake. Fruit consumption increased slightly only in the VC group (23.9% pre vs. 45.5% post). Participation decreased through the weeks: FB (week 2), IG (week 3), and VC (week 4). Retention was higher in the VC (48%) and control (60%) groups. Internet-based interventions employing interactive platforms such as virtual campus can be effective in enhancing participants’ dietary habits in a clinically relevant manner.

## 1. Introduction

Through the last few decades, dietary habits have changed dramatically due to globalization and variations in current lifestyles [1]. Nowadays, traditionally Mediterranean Diet (MD) countries, such as Spain, present a similar dietary pattern to a Western one, according to the Spanish food consumption panel [2]. Data from 2021 display a low consumption of fruit and vegetables (FV) (three servings: two fruit and one vegetable vs. Spanish and the MD recommendations of at least five servings a day), legumes, and nuts [3,4,5]. On the other hand, consumption of dairy, red meat, snacks, soda drinks, and sweets, typical of a Western pattern, is much higher than recommended. This kind of diet involves an elevated intake of energy, saturated fat, salt, and additives, but a low intake of fiber, minerals, and vitamins, which agrees with the findings of our research group (unpublished data of “U. Valladolid healthy survey”, 2018). In this study, we analyzed a sample from the community of the University of Valladolid (U. Valladolid), showing that 64% of the volunteers registered an intake of less than three servings of fruits (serving = 100–150 g) a day and 49% did not eat two servings of vegetables (serving = 200 g) a day.

There is strong evidence that bad eating habits are related to a high BMI [6,7,8]. In Spain, data from the Health Ministry of 2020–21 manifest a prevalence of obesity of 16% among the adult population (16.5% male and 15.5% female) [9]. When considering obesity and overweight together, 53.6% of the adult population have an excess of weight, which is an enormous costly public health matter [6,10]. Comparing national data with our community of U. Valladolid, our research group found a rate of 21% overweight and 3% obesity (“U. Valladolid Healthy Survey”, 2018), which is below the national rate, but still an alarming health issue. Indeed, after the COVID-19 pandemic, eating habits have globally worsened [11,12]. In Spain, many studies indicate that a considerable part of the population gained weight [13] during the lockdown due to a lack of exercise and an increase in the intake of sugar-sweetened beverages, alcoholic beverages, and snacks [14].

An unhealthy diet not only has an impact on weight but also is associated with a high risk of developing chronic non-communicable diseases (NCDs), according to the WHO [6]. On the contrary, a healthy dietary pattern, such as the MD [15,16,17], is related to reducing the risk of death from all causes [18], lowering blood pressure [19], and preventing both cardiovascular disease [20,21,22] and conditions like cancer [23,24]. Also, the MD involves a more sustainable diet by promoting aspects such as biodiversity, seasonality, culinary activities, and traditional, local, and eco-friendly food products [25,26,27].

The WHO recommendations prioritize FV consumption in order to protect against NCDs [28]. In an isolated way, FV intake is related to beneficial effects in preventing coronary disease [29,30], some types of cancer, and mortality [31,32]. Therefore, increasing the consumption of FV in a community is an excellent nutritional educational objective.

Although, as has been mentioned, the pandemic situation has contributed to worsening eating habits, it has also provided an opportunity at a technological level. The use of technologies and social media has increased in the nutrition education field, as well as in the educational field in general, since it was the only way to communicate between communities while keeping security measures imposed [33,34].

Social media (SM) is now a widely used tool in health interventions. It can perform multiple functions such as delivering health information to audiences [35], supporting interaction with the audience in two-way communication (institutions and health professionals) [36] and favoring peer support group discussion [37,38]. Lately, social media has been used to motivate health behavior, which encourages participants to share their own progress, set group challenges, and engage in health behavior competition with peers [39]. The current evidence of the efficacy of social media use in improving diet quality, according to a recent Cochrane systematic review, is low when compared to non-interactive intervention programs [40]. Although the confidence in the evidence is still low, further research in this field, and, also, more protocols and methodology rigor are warranted [41]. However, there is slightly higher evidence for other healthy behaviors such as increasing physical activity in programs using social media platforms, especially Facebook [40]. This indicates that more interventions in the nutrition education field are needed to corroborate whether social media platforms such as Facebook or Instagram, which are widely used among young adults, could be effective in improving the dietary habits of populations.

A university setting is a perfect scenario for the implementation of a nutrition education program since it is both a workplace and a study center [42]. It combines different types of users: students (young adults), professors (adults), and university workers (adults). For this reason, the programs implemented must be adapted to this particular audience [43]. Moreover, it is a place where many of its users spend a lot of time, so they usually have one or even two meals. Also, it is very common to snack, which usually involves high-sugar- and high-fat-content food consumption [44,45]. Our research group found that both brunch and snacks were the principal occasions of food consumption on campus for all U. Valladolid members (“U. Valladolid Healthy Survey”, 2018). And, since the FV intake of both college students and adults is below recommendations [46], this offers a great opportunity to propose a nutrition educational program to increase the FV consumption of this community.

### Objective

The current paper aims to evaluate the effectiveness of an intervention program (“The U. Valladolid Community Eats Healthy” (UVEH)), based on the Theory of Planned Behavior, to increase FV intake in adults from the University of Valladolid, employing three different online methodologies. Secondary objectives were to assess the participation in and adherence to the program according to the different methodologies employed.

## 2. Materials and Methods

### 2.1. Study Design

This study used a randomized controlled trial design to increase the consumption of fruits and vegetables in a university community, employing three different online methodologies: virtual campus (VC), Facebook (FB), and Instagram (IG). In order to use different methodologies, the sample was randomly divided into four groups (VC, FB, IG, and control) by a random sequence generated using the Microsoft Excel program v. 365; three groups took the nutrition educational program, and the other one was a control group. Data were collected at two points: before the beginning of the program (T0) and at the end of it (T1). The trial was registered at ClinicalTrials.gov with the identifier number NCT05900739. The study followed the ethical standards recognized by the Declaration of Helsinki and was approved by the Ethics Committee of the Health Area Valladolid East (Resolution No. PI 22-2632; 10 March 2022). The project was explained to participants before the start (by e-mail or private message), and informed consent was obtained from all participants. To enroll in the program, participants were informed of the group they were assigned to, IG, FB, VC, or control, and were given an enrolment key. The key only gave access to the virtual campus section, placed in the U. Valladolid Moodle platform v.4.0., of their group (IG, FB, VC, and control); it was blind to the existence of other groups and sections.

### 2.2. Participants and Recruitment

The target population of the study was composed of the whole community of the University of Valladolid. The sample size was calculated with the finite population sampling formula, with a 90% level of confidence and a 6% error rate; a sampling plan of 185 participants was estimated. To prevent possible withdraws, the sample size was increased to reach 200 participants. The inclusion criteria were being part of the U. Valladolid community at the time of recruitment, having an IG and/or FB account if they were assigned to those groups, and having made the self-registration to the Moodle virtual campus of each group during the enrolment period. Participants were recruited voluntarily by mailing, which included an infographic explaining the nutritional educational program, which was sent by the virtual department of the U. Valladolid.

### 2.3. Intervention

Both the intervention and control programs were implemented for seven weeks. To make the program more effective, it was theory-based in design, based on the theory of planned behavior (TPB) [47] (Table S1). The contents of the program are summarized in Table 1. 

The intervention in the three platforms had the same topics, although the different activities were adapted to the characteristics of them:

- Virtual campus: weekly webinars that were recorded for consultation. Also, documents such as menu planning sheets, and shopping list models were provided.
- Instagram: videos, posts, stories, and Instagram Lives as workshops (recorded).
- Facebook: videos, posts, stories, and Facebook Lives as workshops (recorded).

The topics, activities, and schedule of the program, in detail, are provided in the Supplementary Materials (Table S2). To encourage participation, each week, a challenge was proposed, so participants could put in practice the information presented during the week; each challenge had a different goal and punctuation. All groups undergoing the program had a section in a Moodle course entitled “The U. Valladolid community eats healthy”, which was employed to enroll in the program and to participate in the challenges. To complete the tasks, participants were asked to send a picture of the recipes proposed through the VC; then, researchers put them nameless back onto the VC or IG and FB accounts, so all participants could vote. The three pictures more voted received marks as follows: the first one received 50%, the second one 30%, and the third one 20%. A ranking was updated every week, so at the end of the program, the winners of each group (VC, FB, and IG) received an e-book about the benefits of a plant-based diet.

### 2.4. Control Group

In the control group, only information about the Mediterranean Diet was provided without interaction or intervention. The contents of the control group program are summarized in Table 1. The control group also had a section in the Moodle course, where, each week, the contents for participants were uploaded. Sections were blind to the rest of the groups; participants could only see and access the section of their group (VC, FB, IG, or control).

### 2.5. Measures

#### 2.5.1. Primary Outcomes: FV Intake

FV consumption was measured using the Mediterranean Diet Adherence Screener (MEDAS-14) questionnaire from the Predimed Study [48], which is validated for the Spanish population, before the intervention (T0) and after it (T1). This questionnaire has a total of 14 items counting each of them as one point; it catalogues the adherence to a Mediterranean dietary pattern as follows: ≤5 points low adherence, 6–9 points medium adherence, and ≥10 high adherence. Items express food groups in servings/day or servings/week, according to Spanish dietary recommendations. Food groups that positively score are fruits, vegetables, legumes, nuts, whole grains, fish, red wine, and vegetable fat from olive oil; on the contrary, food groups that negatively score are saturated fats (butter and margarine), soda drinks, red meat, and sweets. There are two items (nº 3 and 4) that refer to vegetable and fruit intake. To score in item 3, there must be an intake of 2 or more servings/day of vegetables (portion size 200 g), and, in item 4, there must be an intake of 3 or more servings/day of fruits (portion size 100–150 g). The questionnaire was completed through an online form using the application Microsoft Forms from v.365. Microsoft.

#### 2.5.2. Secondary Outcomes

Participation in the program was evaluated using challenge rankings. Also, social demographic outcomes, health conditions, BMI (self-reported), and toxic habits were registered. Data were collected using an online form proposed by the researchers.

### 2.6. Data Analysis

Statistical analyses were made using IBM-SPSS 20.0. Categorical variables were described as absolute (*n*) and relative (%) frequencies, and quantitative variables as the mean (SD). Data normality for quantitative variables was evaluated with the Kolmogorov–Smirnov test (total sample) or Shapiro–Wilk test (groups). The initial equivalence of the groups was assessed with the Kruskal–Wallis test for quantitative variables and the chi-squared test for categorical variables. Intervention effectiveness was analyzed with the W of Wilcoxon test for quantitative variables, and McNemar and Q of Cochran tests for categorical variables. A significance level of *p* < 0.05 was assumed.

## 3. Results

### 3.1. Recruitment

**Figure 1:**
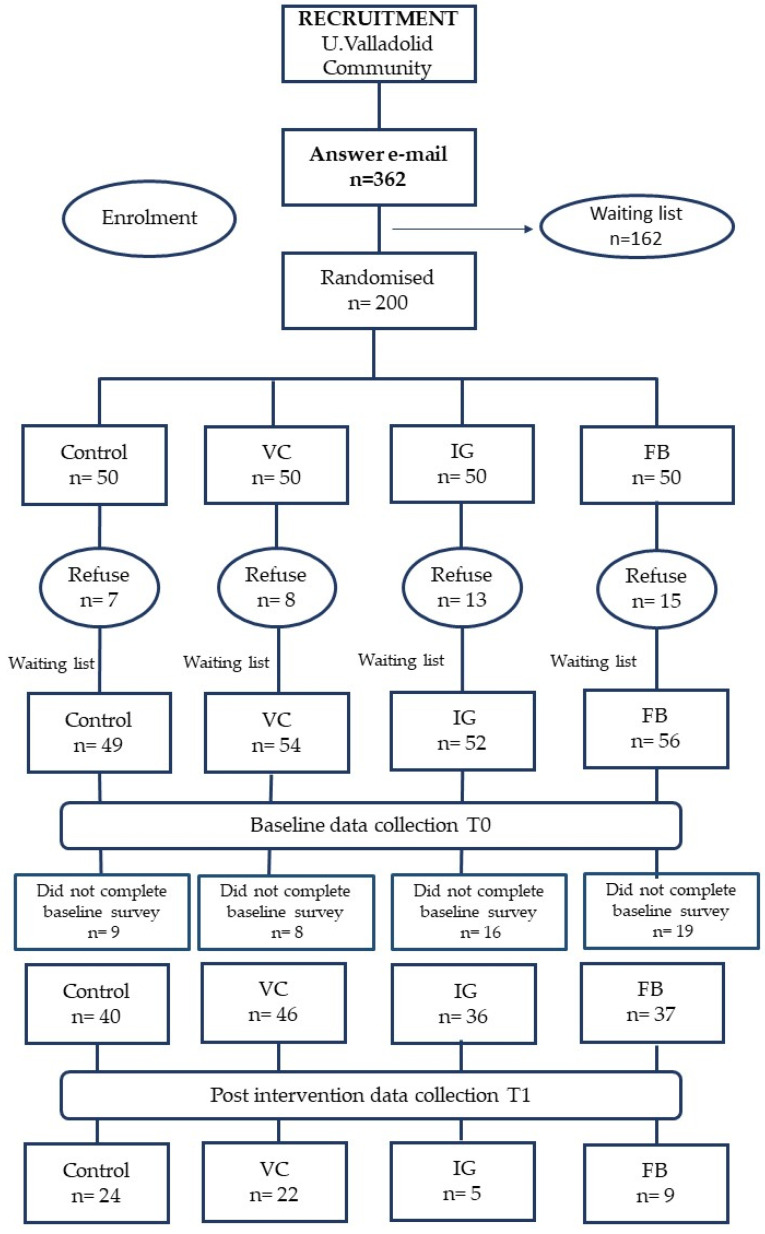
Flowchart of participants from recruitment to post-intervention data collection. U. Valladolid (University of Valladolid), VC (virtual campus), IG (Instagram), and FB (Facebook).

A total of 362 members of the U. Valladolid community responded to the email inviting them to participate in the program (Figure 1). All volunteers were registered by name and email, as they were assessed for eligibility. The first 200 were randomly assigned to four groups: three interventions (VC, FB, and IG) and one control. The first enrollment period lasted for a week. During this time, participants received a key to enroll in the Moodle course “The U. Valladolid community eats healthy”. Additionally, those assigned to FB or IG groups received invitations to join the private accounts on FB and IG named “The U. Valladolid community eats healthy”, as well. Some volunteers declined to participate during the first enrollment period, with notable refusals occurring in both FB and IG groups (*n* = 15 and *n* = 13, respectively). The primary reason for not participating was disagreement with joining a course offered by the university on a platform external to the virtual campus. During the second enrollment period, which lasted another week, invitations were sent to participants on the waiting list to complete the groups, following the randomized sequence initially generated. In this second period, the groups were completed, and, in fact, some volunteers from the first period changed their minds and ultimately joined the program, resulting in a final sample size of 211 participants.

### 3.2. Participants at Baseline

One hundred and fifty-nine participants completed baseline data collection. Demographic characteristics of the baseline sample are shown in Table 2. The mean age of participants was 36.9 (15.3) years, and the percentage of male participants was 24.8%. The nutritional status of the sample, according to BMI, was 24.2 kg/m^2^ (4.1), catalogued as a healthy weight; however, a third of the sample (32.5%) presented an excess of weight. The final sample included more students (51.6%) than administration staff (24.5%) and professors and researchers (23.9%), reflecting the distribution of work activity at the University of Valladolid. Regarding the distribution of U. Valladolid members through the four groups, there was a statistically significantly higher percentage of administration staff in the VC compared to the IG (*p* = 0.024) and control (*p* = 0.020) groups, and, also, a statistically significantly higher percentage of professors and researchers in the control compared to the FB (*p* = 0.033) group. Most participants worked or studied in areas (86.2%) other than the health sciences (13.8%). The majority of participants were healthy (67.3%). A small percentage smoked (5.7%), and most of the participants drank alcohol (72.3%). The principal kind of alcohol consumed was wine or beer (89.6%), with an occasional frequency (57%). Most participants lived with family (64.2%).

The mean Medas-14 score of participants was 6.73 points (1.9), being catalogued mainly as medium adherence to the Mediterranean Diet (62.3%).

There were no statistically significant differences in age, sex, area of knowledge, BMI, and Predimed Score between the groups at baseline. 

### 3.3. Intervention Effectiveness

The results of the questionnaire Medas-14 from the Predimed study to evaluate adherence to the Mediterranean Diet are shown in Table 3. The mean score of the questionnaire was higher in the total sample and in all groups after the intervention. Although there were no statistically significant differences in any of the groups, in VC, there was a notable increase of 1.14 points (*p* = 0.067) compared to the other groups. To assess the effectiveness of the intervention, the evaluation of items 3 and 4 (vegetable and fruit intake, respectively) was performed. The study of these items showed that after the intervention, the intake of vegetables (>2 servings/day, size portion 200 g) was statistically significantly higher in the VC group (17.4% pre vs. 72.7% post); although there were no statistically significant differences in the other groups, there was a higher intake of vegetables after intervention in all of them. Item 4, fruit intake (>3 servings/day; 100–150 g size portion), was slightly higher in the total sample after intervention (23.3% pre vs. 31.7% post), and this could be due to the VC group (23.9% pre vs. 45.5% post) since there were nearly no differences in the intake of fruit in the rest of the groups. In addition to the study of items 3 and 4, the analysis of the rest of the items in the Predimed questionnaire before and after intervention showed no differences except for in item 2 in the VC group. This item refers to the intake of olive oil (>4 spoons of olive oil/day) and it was significantly lower (*p* = 0.03) after the intervention in the VC group.

### 3.4. Participation and Retention

**Figure 2:**
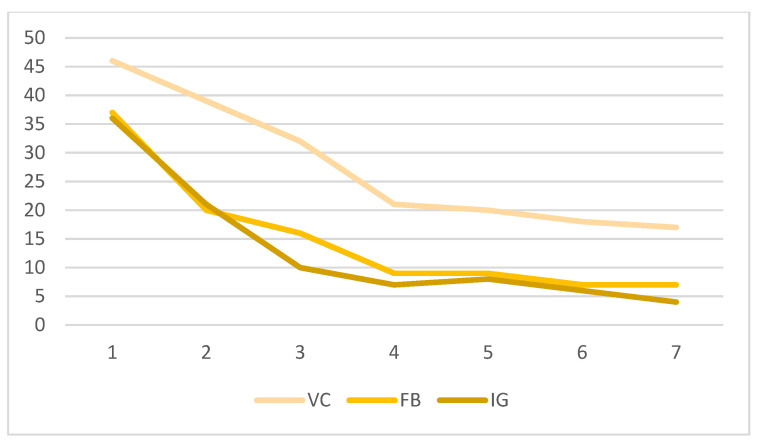
Participation in challenges along the program of the three groups of intervention. Virtual Campus (VC), Facebook (FB), and Instagram (IG).

Figure 2 displays the participation through the program UVEH. Each week, a challenge was proposed to put into practice the contents explained. The VC group showed higher participation and adherence to the program. The FB and IG groups showed decreasing participation. In the FB group, participation decreased earlier than in the IG group, and, at the end of the program, participation in these groups was much lower than in the VC group.

When considering the different activities proposed in the FB and IG groups, the livestreams that took place in weeks 4 and 7 received a great proportion of interactions (45.5% FB and 29.4% IG). These groups also used a very similar methodology to the one employed in the VC group.

The retention rate of participants was as follows: VC 48%, FB 24.3%, IG 14%, and control 60%. This again indicated a higher adherence to the program in the groups that employed the Moodle platform compared to social media groups.

## 4. Discussion

The present study evaluated the efficacy of a nutritional intervention program to increase the intake of FV among adults from a university community, applying three different online methodologies: VC, FB, and IG. Regarding vegetable intake, the results showed a statistically significant increase in the VC group (17.4% pre vs. 72.7% post). In the other intervention groups, there was also an increase in vegetable intake; however, it was not statistically significant. It is worth noting the small size of the post-intervention groups, FB (*n* = 5) and IG (*n* = 9), which may have contributed to these results. The other variable studied was fruit intake, which increased in the total sample after the intervention, primarily driven by the VC group (23.9% pre vs. 45.5% post). However, no differences were found in the rest of the intervention groups.

There have been widely reported interventions to increase FV intake in children. In this field, the effective interventions were those that provided free FV [49], the ones that increased in-school availability [50], strategies that targeted the home and family environment [51], and computer-based and SMS-delivered interventions [52]. Generally, there is a greater increase in fruit intake compared to vegetables in children [53], which contrasts the findings of this paper. However, it should be noted that the target population of this study was young adults and adults, among whom there might not be a preference for fruits over vegetables as seen in children. In this regard, a meta-analysis conducted by Masoumeh J. et al. [54] showed that community-based interventions in adults led to a significant increase in vegetable intake and a non-significant increase in fruit consumption. This meta-analysis also indicated that face-to-face interventions were more effective than internet-delivered ones in increasing FV aggregated consumption, which aligns with the results of the present study, especially when we equate VC intervention to in-person, even though it was conducted through an online platform Additionally, interventions lasting less than 24 weeks were more effective than those lasting 24 weeks or more, which is consistent with the findings of this study, where a 7-week intervention period resulted in increased FV consumption in all groups compared to baseline values. A notable aspect of comparing the results of the mentioned studies with the present one is the serving size of FV. While the WHO recommends portion sizes of 80–100 g for fruits and vegetables (five a day) [28], the present study used portion sizes of 200 g for vegetables and 100–150 g for fruit, which are the portion sizes expressed in items nº 3 and 4 of the MEDAS-14 questionnaire [48], following the MD and Spanish dietary recommendations [3,4,5]. Therefore, the increase of one serving of vegetables in the VC group is not only statistically significant but also clinically relevant, as it translates to an intake of 400 g of vegetables per day.

Another relevant aspect of the present study is the different methodologies employed and their effectiveness in increasing the intake of FV. When comparing face-to-face interventions vs. web-based ones, Al-Awadi et al. [55] found that interventions with personal interactions were more effective in improving dietary changes, such as higher FV consumption, than those delivered by web (e-mails and web, basically). This is similar to the results of this paper, where the group with more one-to-one interaction (VC) presented better results in increasing FV consumption. In this regard, the methodology of this study presents an affordable advantage compared to face-to-face and personal interventions, which is the low cost of the VC achieved by employing an online platform, but maintaining a high interaction rate. Another systematic review conducted by Livingstone et al. [56] concluded that digital interventions were poorly effective in increasing vegetable intake in adults; however, most of the studies reviewed used SMS, apps, and websites, which does not correspond with the online methodologies employed in this study. Two of the intervention groups presented in this study employed SM (FB and IG) to increase FV intake in adults. Lately, the uses of SM for health purposes have been widely studied [39], and when it comes to health interventions, SM use seems effective in increasing health knowledge, reducing risky behaviors, and adopting health behaviors. Also, SM use by patients can improve their literacy of their condition when following SM accounts of recognized health institutions or recognized clinicians. SM can be used, as well, for peer groups and advocacy between patients with the same condition [57]. However, there is a issue with the use of SM in health interventions, which is due to the lack of engagement of participants in the long term [58]. This is perfectly aligned with the results of the present study, where the FB and IG groups showed a decrease in participation much sooner than in the intervention groups that employed a more familiar platform to the U. Valladolid community: VC and control. Also, in SM groups, the retention rate was much lower than in both the VC and control groups. The VC group was the one that showed the highest rate of participation in the weekly challenges, with a drop peak at Week 4. In social media groups, IG and FB, the drop peaks appeared before, at Week 3 and 2, respectively. Additionally, the retention rate was higher in the groups with implementation through the Moodle platform: VC 48% and control 60%. It should be highlighted that in both VC and control groups, there was a higher percentage of administration staff (VC vs. IG (*p* = 0.024)) and professors (control vs. FB (*p* = 0.033)) who were older participants, usually more motivated to join training programs and voluntary interventions, which may have contributed to these results. Also, it seems that the use of the Moodle platform, which the U. Valladolid community is very familiar with, provided a higher engagement rate. The lower retention and participation rates of FB and IG groups may be due to the large number of students in them, which could seem surprising since these SM platforms are very commonly used among young adults. Moreover, it seems that the typical use of social media by young adults is primarily for connecting with friends or family, entertainment, purchasing clothes or other products, and following famous individuals. Therefore, the results of this study support the need for further research on the use of social media for nutritional interventions to address the challenge of sustaining participant engagement over the long term. While social media use for patients appears promising, its effectiveness in nutritional education among healthy populations is less evident.

The present study has several strengths and limitations. The main limitation was that all participants were volunteers, and all were accustomed to using the Moodle platform, but not all were familiar with the use of Facebook or Instagram. This lack of familiarity impacted the engagement and participation of these groups, resulting in a very small final size for these two groups. The small sample size complicates the achievement of adequate statistical power to detect significant differences among the groups.

In terms of strengths, a notable highlight is the demonstration that internet-delivered interventions utilizing interactive platforms, such as virtual campuses, can effectively improve participants’ eating habits, specifically by increasing fruit and vegetable intake in a clinically relevant manner. 

## 5. Conclusions

Internet-based interventions employing interactive platforms such as virtual campus by the Moodle platform, but not SM, have demonstrated effectiveness in enhancing participants’ dietary habits. This innovative application of such platforms, commonly used for educational purposes, is very promising due to its affordability and its potential for wide reach. 

Additionally, the effectiveness of theory-based interventions with a planned protocol should be highlighted in the nutritional educational field.

## Figures and Tables

**Table 1:** Topics of the intervention and control groups.

	Intervention	Control
Week 1	Presentation of the program. Healthy eating	Healthy eating
Week 2	FV: nutritional value, types, seasonality, benefits for health	MD—principal foods
Week 3	Shopping FV: tips, places, conservation methods	MD—a lifestyle
Week 4	Healthy menu planning with “My Plate”	MD—pyramid
Week 5	How to cook FV avoiding food waste	Health benefits of the MD
Week 6	Healthy snacks using FV	How to practice the MD on a daily basis
Week 7	Batch cooking	Myths of the MD

MD: Mediterranean diet; FV: fruits and vegetables.

**Table 2:** Demographic characteristics of participants at baseline as total sample and according to the intervention group.

	Total Sample (*n* = 159)	VC (*n* = 46)	FB (*n* = 37)	IG (*n* = 36)	Control (*n* = 40)
***Gender***					
Female	75.2%	84.8%	62.2%	80.6%	72.5%
Male	24.8%	15.2%	37.8%	19.4%	27.5%
Age (years) mean (SD)	37.1 (15.2)	40.1 (13.6)	36.1 (15.3)	34.5 (16.4)	36.2 (16.2)
***Nutritional status***					
BMI (kg/m^2^) mean (SD)	24.2 (4.1)	23.6 (3.7)	24.1 (3.7)	24.9 (4.0)	24.4 (4.9)
Underweight	0.6%	6.7%	2.7%	0	2.5%
Healthy weight	63.3%	64.4%	70.3%	55.6%	62.5%
Overweight	21.5%	20%	16.2%	30.6%	20%
Obese	12%	8.9%	10.8%	13.9%	15%
***Work activity***					
Student	51.6%	37%	62.2%	58.3%	52.5%
Administration staff	24.5%	41.3% *	27%	13.9%	12.5%
Professors and researchers	23.9%	21.7%	10.8%	27.8%	35% ^†^
***Living situation***					
With family	64.2%	67.4%	70.3%	55.6%	62.5%
With friends	25.2%	21.7%	21.6%	30.6%	27.5%
Residential college	0.6%	0	0	0	2.5%
Own home	10%	10.9%	8.1%	13.9%	7.5%
***Area of knowledge***					
Health Science	13.8%	10.9%	18.9%	11.1%	15%
Other	86.2%	89.1%	81.1%	88.9%	85%
***Toxic habits***					
Smoking	5.7%	2.2%	8.1%	5.6%	7.5%
Alcohol drinking	72.3%	69.6%	73%	72.2%	75%
***Health conditions***					
HBP	5.7%	6.5%	5.4%	5.6%	5%
Dyslipidemia	8.8%	13%	8.1%	5.6%	7.5%
Arthritis	6.9%	8.7%	16.2%	0	2.5%
No conditions	67.3%	65.2%	64.9%	75%	65%
**Medas-14 Questionnaire**					
Predimed Score mean (SD)	6.73 (1.9)	6.61 (1.8)	6.49 (2.0)	6.78 (2.2)	7.1 (1.8)
Low adherence	29.6%	32.6%	35.1%	30.6%	20%
Medium adherence	62.3%	65.2%	54.1%	54.1%	70%
High adherence	8.2%	2.2%	10.8%	11.1%	10%

VC (virtual campus), FB (Facebook), IG (Instagram), BMI (body mass index), and HBP (high blood pressure). * VC vs. IG (*p* = 0.024) and control (*p* = 0.020). ^†^ Control vs. FB (*p* = 0.033).

**Table 3:** Medas-14 Questionnaire: mean score, item 3 (vegetable intake), and item 4 (fruit intake) before and after intervention for total sample and intervention groups.

	Total Sample	VC	FB	IG	Control
**Medas-14 Questionnaire**					
Pre-intervention mean (SD)	6.73 (1.9)	6.61 (1.8)	6.49 (2.0)	6.78 (2.2)	7.10 (1.8)
Post-intervention mean (SD)	7.62 (1.9)	7.75 (1.9)	7,11 (1.3)	7.40 (3.0)	7.75 (1.9)
**Item 3 Vegetable intake** **(>2 servings/day; 200 g)**					
V intake pre-intervention	24.5%	17.4%	21.6%	27.8%	32.5%
V intake post-intervention	38.3%	72.7% *	33.3%	40%	40%
**Item 4 Fruit intake** **(3 or more servings/day; 100–150 g)**					
F intake pre-intervention	23.3%	23.9%	24.3%	19.4%	25%
F intake post-intervention	31.7%	45.5%	22.2%	20%	25%

VC (virtual campus), FB (Facebook), IG (Instagram), V (vegetables), and F (fruit), * *p* < 0.05.

## Data Availability

The original contributions presented in the study are included in the article/Supplementary Materials, further inquiries can be directed to the corresponding author/s.

## Supplementary Materials

The following supporting information can be downloaded at https://www.mdpi.com/article/10.3390/nu16091308/s1, Table S1. Determinants of the theory of planned behavior (TPB), behavior change technique, and activities in the three intervention groups; Table S2. Topics, schedule, and activities adapted to each platform of the program “The U. Valladolid community eats healthy”.
