# Supplementary material for: Social Media for Nutrition Education—A Randomized Controlled Trial to Promote Fruit and Vegetable Intake in a University Setting: “The University of Valladolid Community Eats Healthy” Study
Source: Nutrients. 2024 Apr 26;16(9):1308. doi: 10.3390/nu16091308 (PMC11085167; doi:10.3390/nu16091308)
Supplement: Supplementary file 1 [file nutrients-16-01308-s001.zip › nutrients-2949468-supplementary.pdf]

SUPLEMENTARY MATERIAL

**Table S1.** Determinants of theory of planned behaviour (TPB), behaviour change technique and activities in the three intervention groups.

| Determinants of TPB | Behavior change technique | Activities<br>VC | IG | FB |
|---|---|---|---|---|
| Attitude | Provide information about health benefits of eating FV. | Webinar 1: “Healthy eating”. | – Video “healthy eating”. Post- FV servings, FV consumption and health benefits. | – Video “healthy eating”. Post- FV servings, FV consumption and health benefits. |
| | Explaining the types of FV, its nutritional value, seasonality, places to purchase them. | Webinar 2 and 3: ”FV best partners of your health” and “How to be a responsible consumer”. | – Posts: nutritional value of FV, places to buy FV, seasonal calendar of FV, places to buy FV and tips for FV conservation, to be a responsible consumer. | – Posts: nutritional value of FV, places to buy FV, seasonal calendar of FV, places to buy FV and tips for FV conservation, to be a responsible consumer. |
| Subjective norms | Propose healthy snacking ideas | Webinars 5 and 6: “Culinary techniques for FV” and “Healthy snacking ideas with FV”. | – Posts about FV snacks and recipes.<br>– Videos of culinary techniques for FV and healthy snacking. | – Posts about FV snacks and recipes.<br>– Videos of culinary techniques for FV and healthy snacking. |
| Perceived behavioral control | Work on abilities such as healthy menu designing, batch cooking techniques, grocery list development | – Webinars 5 and 3: “Culinary techniques for FV” and “How to be a responsible consumer”.<br>– Webinars 4 and 9: “My Plate: Healthy menu planning” and “Batch cooking”. | – IG Live: “How to plan a healthy menu”.<br>– IG Live:” Batch cooking”. | – FB Live: “How to plan a healthy menu”.<br>– FB Live: “Batch cooking”. |

VC: Virtual campus, FV: fruit and vegetable, IG: Instagram, FB: Facebook

**Table S2a.** Topic, schedule, platform, activities, and materials for the first week of "The U. Valladolid eats healthy" program during week 1.

| GROUP | TOPIC | IMPLEMENTATION Platform | IMPLEMENTATION Activities | SCREENSHOTS, TOOLS, AND MATERIALS. |
|---|---|---|---|---|
| ***WEEK 1*** | | | | |
| CONTROL | Healthy eating[1] | VC | A document with information about foundations of healthy eating: nutrients, foods, energetic balance, and Mediterranean Diet pattern. | 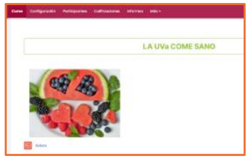  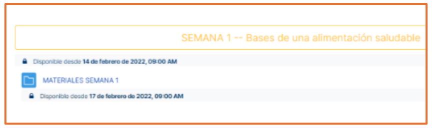  |
| INTERVENTION | Program presentation | VC | - Webinars calendar.<br>- Challenges and punctuation.<br>- Welcome vídeo. | 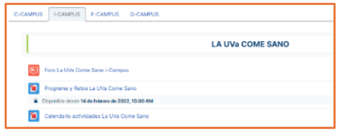  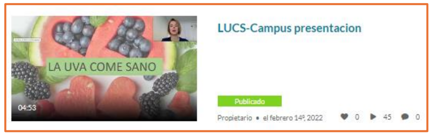  |
| | | IG | – Welcome posts (2)<br>– Post with the weeks and topics of the program.<br>– Post explaining the challenges of each week. | 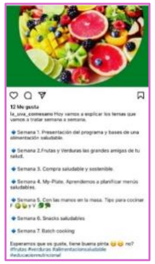 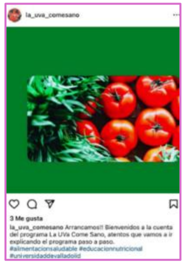 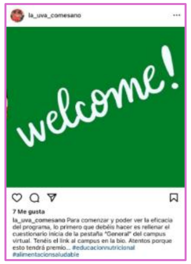  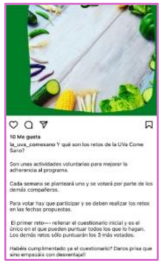 |
| | | FB | – Welcome post.<br>– Post with the calendar of the program.<br>– Post with video explaining the challenges of each week. | 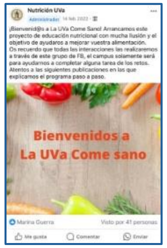  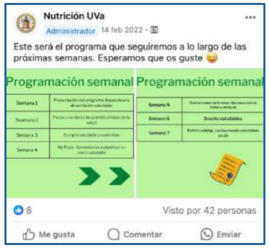  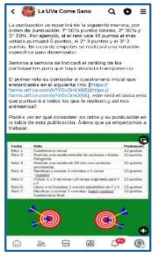 |

| | | |
|---|---|---|
| Healthy eating[1,2,3] | VC | Webinar 1 (recorded) and pdf document with the content: 1. Nutrients / 2. Food and group food / 3. Dietary patterns / 4. Diet and food guides. |

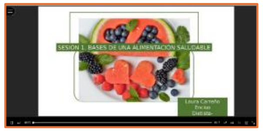
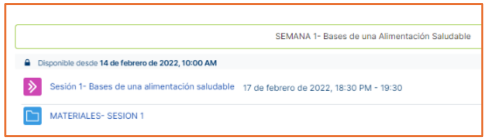


| | | |
|---|---|---|
| | IG | – Post about the objective of the program: to increase FV consumption.<br>– Video about nutrients, food, and health.<br>– Post about food and nutrients classification.<br>– Post about MD pattern<br>– 3 stories to announce the posts and videos. |

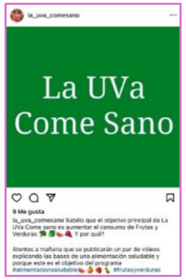

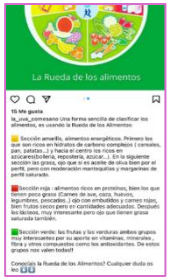

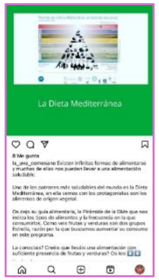

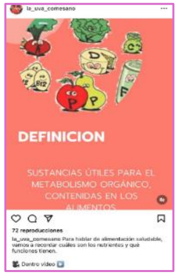


| | | |
|---|---|---|
| | FB | – Post about nutrients and food content in nutrients.<br>– Video about nutrients and foods<br>– Post about food classification<br>– Post about dietary patterns and MD food guide |

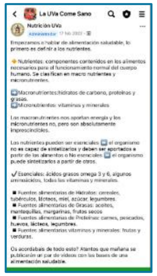
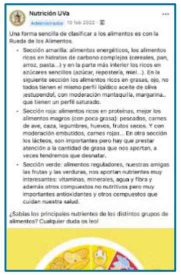
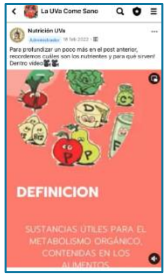

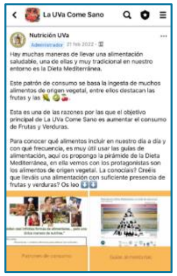

**Challenge nº1** **Complete base-line questionnaire (10 points)**

VC: Virtual campus, IG: Instagram, FB: Facebook, FV: Fruits and vegetables, MD: Mediterranean Diet.

**Table S2b.** Topic, schedule, platform, activities, and materials for the second week of "The U. Valladolid eats healthy" program during week 2.

| GROUP | TOPIC | IMPLEMENTATION Platform | IMPLEMENTATION Activities | SCREENSHOTS, TOOLS, AND MATERIALS. |
|---|---|---|---|---|
| ***WEEK 2*** | | | | |
| CONTROL | MD- principal foods[3] | VC | A document about the principal food characteristics in MD pattern. | 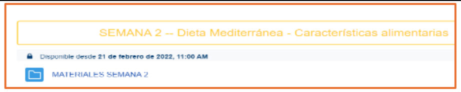 |
| INTERVENTION | FV: health benefits[1] | VC | – Webinar n2 (recorded) about FV benefits, with the content: 1. FV definition / 2. Nutritional value of FV/ 3. Seasonality / 4. Portion size recommended / 5. Health benefits of FV.<br>– Seasonal fruit and vegetable calendar". | 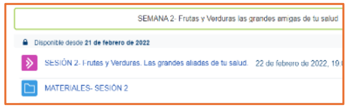 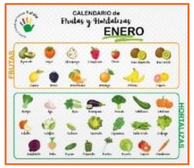 |
| | | IG | – Post about vegetables: types, definition, nutritional content.<br>– Post about fruits: classification, definition, nutritional content.<br>– Post about seasonality and seasonal calendar of fruits and vegetables.<br>– Post about portion size recommended for vegetables.<br>– Post about portion size recommended for fruits.<br>– Post about FV consumption and benefits for health. | 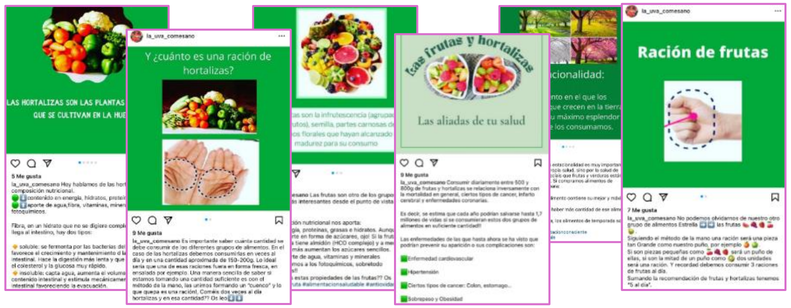 |
| | | FB | – Post about vegetables, types, classification, and its nutritional value.<br>– Post about fruits: types, classification, and nutritional value.<br>– Post with seasonal FV calendar.<br>– Post about seasonality of FV.<br>– Post with video about FV portion size recommended.<br>– Post about health benefits of consuming enough FV. | 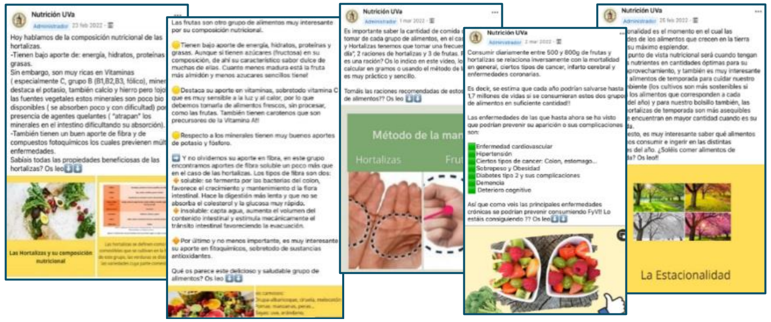 |
| | **Challenge nº2** | **Simple recipe of F or V (10 points)** | | |

VC: Virtual campus, IG: Instagram, FB: Facebook, FV: Fruits and vegetables, MD: Mediterranean Diet.

**Table S2c.** Topic, schedule, platform, activities, and materials for the third week of "The U. Valladolid eats healthy" program during week 3.

| GROUP | TOPIC | IMPLEMENTATION Platform | IMPLEMENTATION Activities | SCREENSHOTS, TOOLS AND MATERIALS. |
|---|---|---|---|---|
| *WEEK 3* | | | | |
| CONTROL | MD-A lifestyle[3] | VC | A document about the non-nutritional characteristics of MD pattern. | 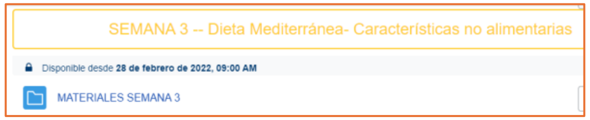 |
| INTERVENTION | Shopping FV: tips, places, conservation methods [1] | VC | – Webinar nº 3 (recorded): "How to be a responsible consumer". Content: 1. Food waste, 2. Responsible consumer (tips before, during and after shopping food), 3. FV conservation.<br>– Shopping list model | 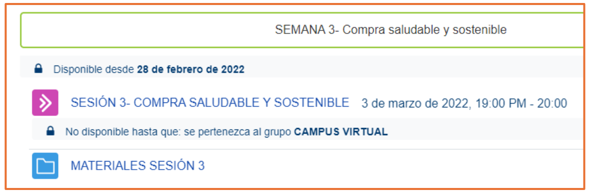 |
| | | IG | – Post with tips of how to be a responsible consumer.<br>– 4 stories about food waste with a questionnaire.<br>– 3 stories about FV conservation. | 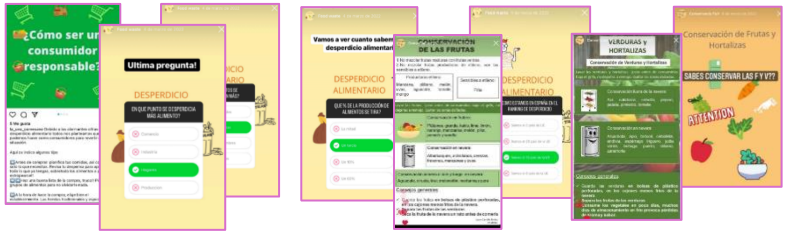 |
| | | FB | – 4 posts with a questionnaire about food waste<br>- Post about food waste, and tips on how to be a responsible consumer.<br>– 2 infographics about FV correct conservation. | 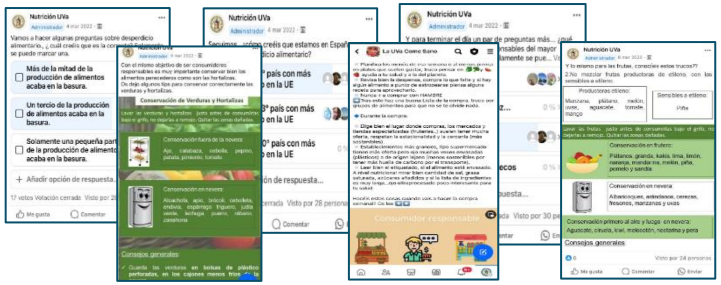 |
| | Challenge nº3 | | Recipe using processed F and/or V (15 points) | |

VC: Virtual campus, IG: Instagram, FB: Facebook, FV: Fruits and vegetables, MD: Mediterranean Diet.

**Table S2d.** Topic, schedule, platform, activities, and materials for the fourth week of "The U. Valladolid eats healthy" program during week 4.

| GROUP | TOPIC | IMPLEMENTATION<br>Platform | <br>Activities | SCREENSHOTS, TOOLS, AND MATERIALS. |
|---|---|---|---|---|
| ***WEEK 4*** | | | | |
| CONTROL | MD-Food guide, MD pyramid [3] | VC | A document explaining the dietary pyramid of MD for Spanish population. | 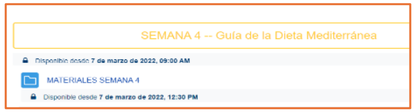 |
| INTERVENTION | Healthy menu planning with "My Plate" | VC | Webinar nº4 (recorded): "My Plate: healthy menu planning". Content: 1. Healthy eating / 2. Food guides / 3. My plate guide / 4 Healthy menu planning. | 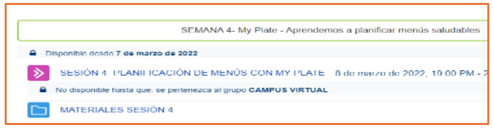 |
| | | IG | – Instagram live: "My Plate: healthy menu planning".<br>– Post with a menu scheme to help with healthy menu planning. | 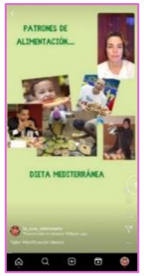 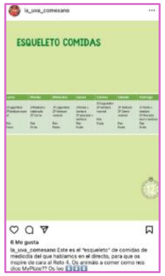 |
| | | FB | – Facebook live: "My Plate: healthy menu planning".<br>– Post with a menu scheme to help with healthy menu planning. | 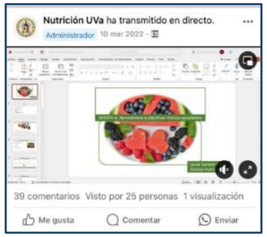 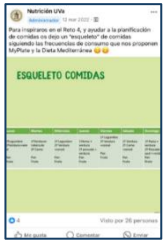 |
| | **Challenge nº4** | **Cooking 3 meals (lunch or dinner) following "My Plate" guide (20 points)** | | |

VC: Virtual campus, IG: Instagram, FB: Facebook, FV: Fruits and vegetables, MD: Mediterranean Diet.

**Table S2e.** Topic, schedule, platform, activities, and materials for the fifth week of "The U. Valladolid eats healthy" program during week 5.

| GROUP | TOPIC | IMPLEMENTATION<br>Platform | <br>Activities | SCREENSHOTS, TOOLS, AND MATERIALS. |
|---|---|---|---|---|
| ***WEEK 5*** | | | | |
| CONTROL | Health benefits of MD [3] | VC | A document explaining health benefits associated with MD dietary pattern. | 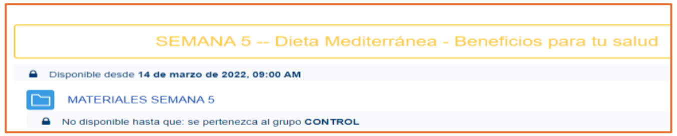 |
| INTERVENTION | Cooking FV: culinary techniques and tips. | VC | – Webinar nº5 :" Hands-on Cooking - Tips for Cooking Fruits and Vegetables". Content: 1. Nutritional value and culinary techniques, 2. Different culinary techniques, 3. Original recipes to cook FV.<br>– Original recipes ideas for cooking FV | 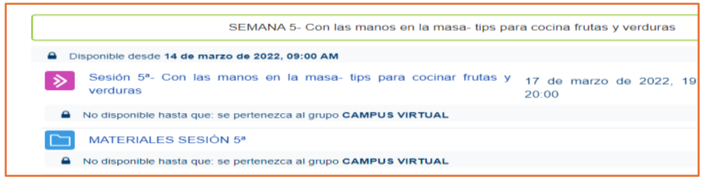 |
| | | IG | – Post about different types of culinary techniques<br>– 2 posts with videos about culinary techniques and nutritional value.<br>– 2 posts with original recipes for FV | 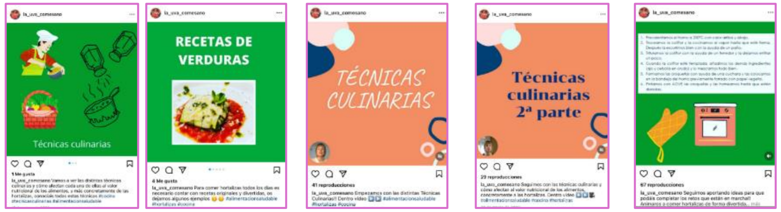 |
| | | FB | – Post about the different culinary techniques most employed.<br>– 2 posts with videos about culinary techniques and nutritional value.<br>– 2 posts with original recipes for FV | 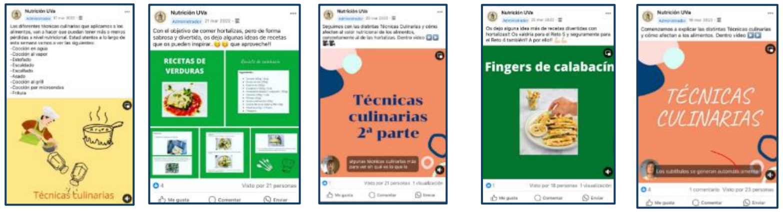 |
| | **Challenge nº5** | **1 or 2 recipes using innovative culinary techniques for FV (25 points)** | | |

VC: Virtual campus, IG: Instagram, FB: Facebook, FV: Fruits and vegetables, MD: Mediterranean Diet.

**Table S2f.** Topic, schedule, platform, activities, and materials for the sixth week of "The U. Valladolid eats healthy" program during week 6.

| GROUP | TOPIC | IMPLEMENTATION<br>Platform | <br>Activities | SCREENSHOTS, TOOLS AND MATERIALS. |
|---|---|---|---|---|
| *WEEK 6* | | | | |
| CONTROL | How to practice MD on a daily basis | VC | A document with an example of a MD menu scheme, and traditional Spanish MD recipes. | 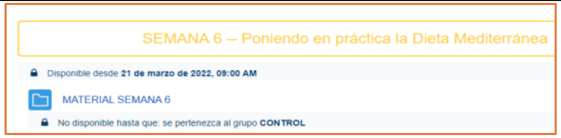 |
| INTERVENTION | Healthy snacking with FV | VC | – Webinar nº6: "Healthy snacking with FV". Content: 1. Daily meals, 2. Breakfast and snacking, 3. Healthy snacks with FV | 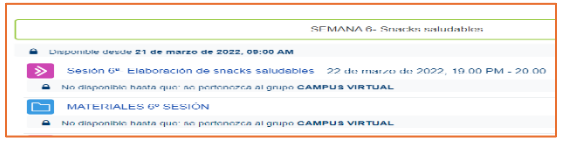 |
| | | IG | – Post about how many meals a day should be recommended.<br>– Post with original recipes for snacks of FV | 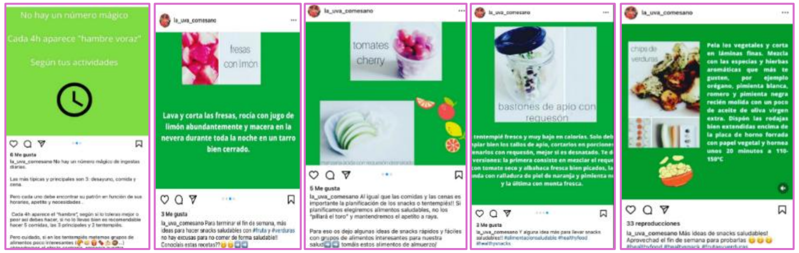 |
| | | FB | – Post about the number of meals a day recommended.<br>– 3 posts with original recipes for snacks of FV | 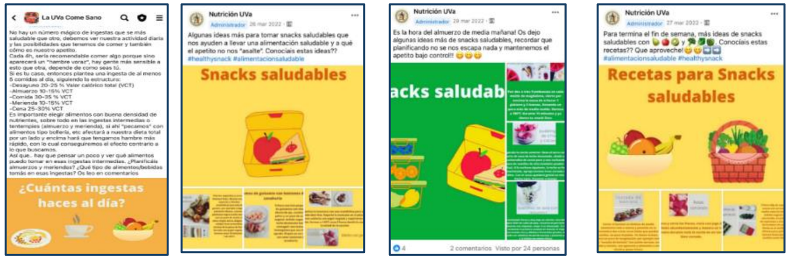 |
| | Challenge nº 6 | **Preparing 3 healthy snacks to take away (25 points)** | | |

VC: Virtual campus, IG: Instagram, FB: Facebook, FV: Fruits and vegetables, MD: Mediterranean Diet.

**Table S2g.** Topic, schedule, platform, activities, and materials for the seventh week of “The U. Valladolid eats healthy” program during week 7.

| GROUP | TOPIC | IMPLEMENTATION<br>Platform | <br>Activities | SCREENSHOTS, TOOLS AND MATERIALS. |
|---|---|---|---|---|
| *WEEK 7* | | | | |
| CONTROL | Myths of the MD | VC | -A questionnaire about myths of MD<br>-A document analysing the myths proposed and debunking them. | 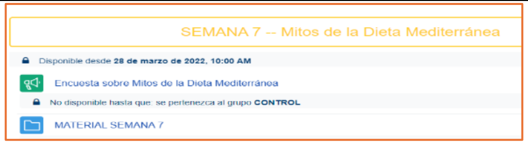 |
| INTERVENTION | Batch cooking: healthy menus for a week | VC | - Webinar nº 7 (recorded): “Batch cooking: healthy menus in 2h cooking”. Content: 1. Definition of Batch cooking: advantages and disadvantages, 2. How to plan a menu for batch cooking, 3. Menus examples for batch cooking. | 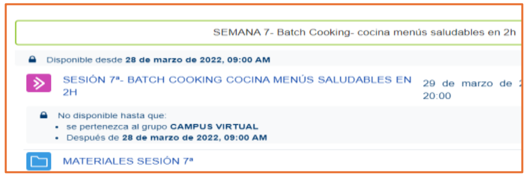 |
| | | IG | - Instagram Live: “Batch cooking: healthy menus in 2h cooking”<br>-Post with video about the benefits of batch cooking<br>-Post with a menu adapted to batch cooking | 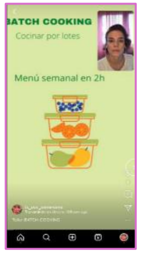 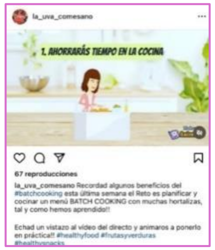 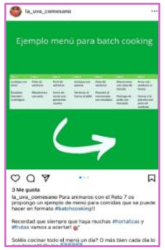 |
| | | FB | -Facebook Live: “Batch cooking: healthy menus in 2h cooking”<br>-Post with video about the benefits of batch cooking<br>-Post with a menu adapted to batch cooking | 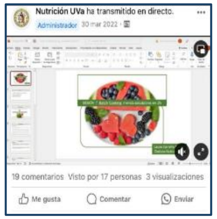 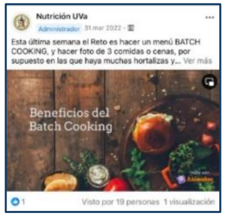 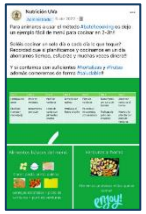 |
| | Challenge nº 7 | **Cooking 3 meals with batch cooking design (30 points) + Completing post-intervention survey (10 points)** | | |

VC: Virtual campus, IG: Instagram, FB: Facebook, FV: Fruits and vegetables, MD: Mediterranean Diet.

1. Agencia Española de Seguridad Alimentaria (AESAN). Recomendaciones dietéticas saludables y sostenibles complementadas con recomendaciones de actividad física para la población española. Come sano, muévete y cuida tu planeta. AESAN; 2022. Disponible en: https://www.aesan.gob.es/AECOSAN/docs/documentos/nutricion/RECOMENDACIONES_DIETETICAS.pdf
2. Aranceta-Bartrina J, Partearroyo T, López-Sobaler AM, Ortega RM, Varela-Moreiras G, Serra-Majem L, Pérez-Rodrigo C; Col-laborative Group for the Dietary Guidelines for the Spanish Population (SENC). Updating the Food-Based Dietary Guidelines for the Spanish Population: The Spanish Society of Community Nutrition (SENC) Proposal. Nutrients. 2019 ;11(11):2675. doi: 10.3390/nu11112675
3. Serra-Majem L, Tomaino L, Dernini S, Berry EM, Lairon D, de la Cruz JN, et al. Updating the mediterranean diet pyramid to-wards sustainability: Focus on environmental concerns. Int J Environ Res Public Health. 2020 ;17(23):1–20
